# Drug-induced subacute cutaneous lupus erythematosus involving osimertinib

**DOI:** 10.1016/j.jdcr.2024.08.032

**Published:** 2024-09-19

**Authors:** Christian L. Bailey-Burke, Carlos H. Nousari, Michael W. Wangia

**Affiliations:** a Duke University School of Medicine, Durham, North Carolina; b Department of Dermatology, University of Miami, Miami, Florida; c Florida Institute of Dermatology, Winter Garden, Florida

**Keywords:** DI-SCLE, drug reaction, EGFR TKI, immunotherapy, osimertinib

## Introduction

Epidermal growth factor receptor tyrosine kinase inhibitors (EGFR TKIs) are the standard treatment for non-small cell lung cancer (NSCLC) patients who harbor the EGFR mutation.^1^ However, despite their initial potent response, first- and second-generation EGFR TKIs often become ineffective after 9-14 months of use, which has led to the development of third-generation EGFR TKIs, including osimertinib. Third-generation EGFR TKI medications have resulted in significantly longer disease-free survival in NSCLC patients.^2^

While proven valuable, EGFR TKIs are not without adverse effects (AEs). Commonly reported AEs range from cutaneous reactions, including skin ulceration and dermatitis, to gastrointestinal events such as stomatitis, nausea, and vomiting.

Amid this array of AEs, one intriguing outcome is the emergence of drug-induced subacute cutaneous lupus erythematosus (DI-SCLE) in response to third-generation EGFR TKIs. There have been 2 case reports of DI-SCLE after initiating the third-generation EGFR TKI erlotinib, with one of the reports showing that DI-SCLE can worsen with subsequent use of osimertinib.^3^^,^^4^ In this report, we discuss a case of DI-SCLE associated with the initiation of osimertinib therapy.

## Case report

**Fig 1:**
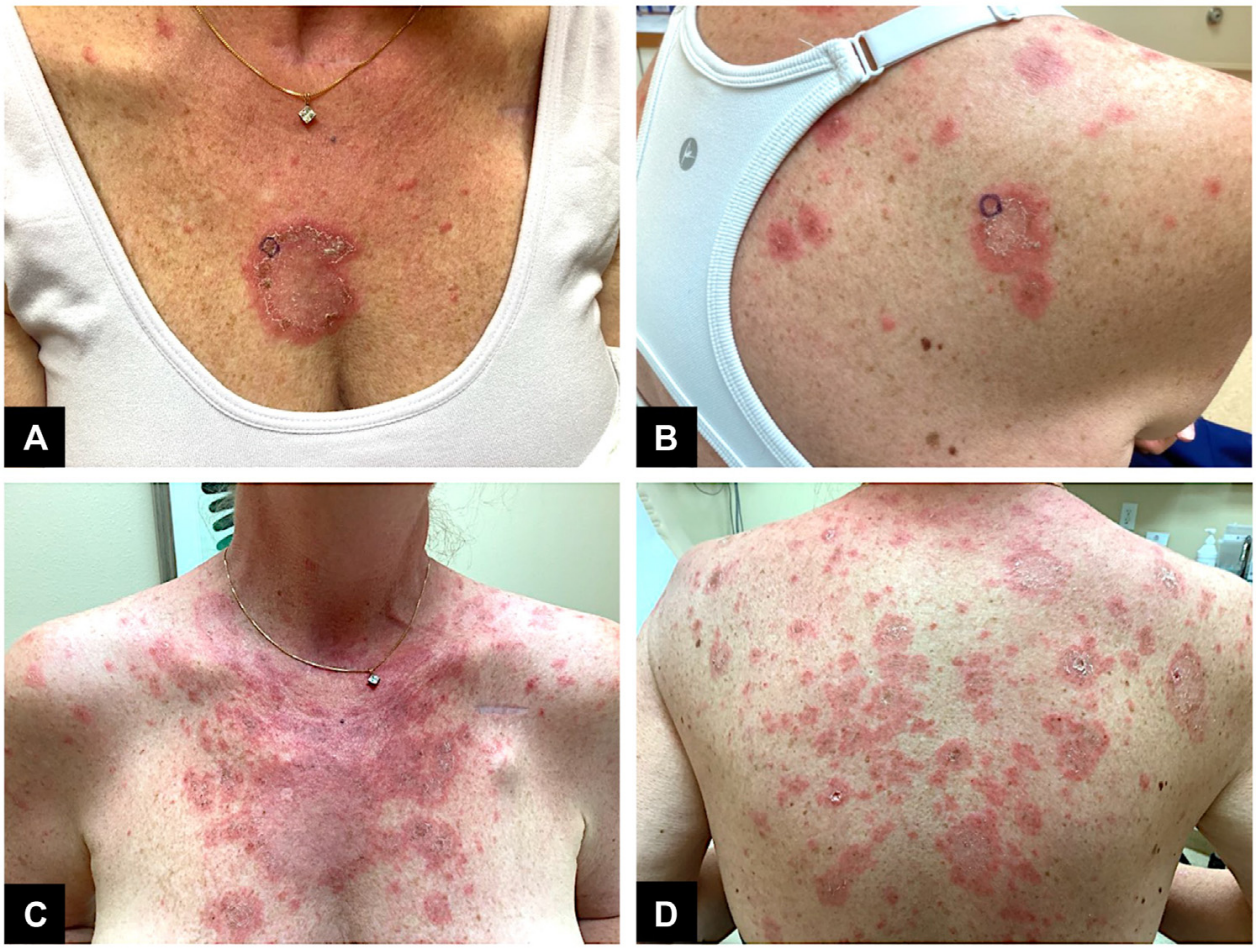
**A,** Patient showing erythematous, annular plaques with peripheral scales on the chest and (**B**) on the right anterior lateral proximal upper arm with associated punch biopsy sites (*circled*) at initial visit. **C** and **D,** illustrate worsening presentation on the chest and upper back, respectively, at 4-week follow-up.

A 51-year-old female treated with osimertinib 80 mg daily for 3 months for stage IVB lung adenocarcinoma presented for worsening bilateral arm and truncal rash that developed within 2 weeks of starting chemotherapy. She denied a history of inflammatory skin disease. She did not have any signs or symptoms of systemic lupus erythematosus. Her medications are notable for bupropion 150 mg, apixaban 5 mg, gabapentin 300 mg, levothyroxine 25 mcg, thyroid tablets (United States Pharmacopeia [USP]) 120 mg, spironolactone-hydrochlorothiazide (25-25 mg), and tirzepatide 5 mg/0.5 mL (Table I). The patient had taken these medications for several years before her presentation. On physical exam, there were polycyclic erythematous plaques with peripheral scale present on the chest, upper back, and arms (Fig 1).

**Table I:** Patient prescription and over-the-counter medications before treatment for DI-SLCE

Medication	Administration	Dosage	Frequency (if known)
Bupropion HCl	Oral	150 mg	q.24
Calcitriol	Oral	0.25 mcg	
Apixaban	Oral	5 mg	
Gabapentin	Oral	300 mg	
Levothyroxine	Oral	25 mcg	
Methylprednisone	Oral	4 mg	
Thyroid tablets, USP	Oral	120 mg	
Spironolactone-hydrochlorothiazide	Oral	25-25 mg	
Tirzepatide	Subcutaneous injector	5 mg/0.5 mL	

*DI-SLCE*, Drug-induced subacute cutaneous lupus erythematosus; *USP*, United States Pharmacopeia.

**Fig 2:**
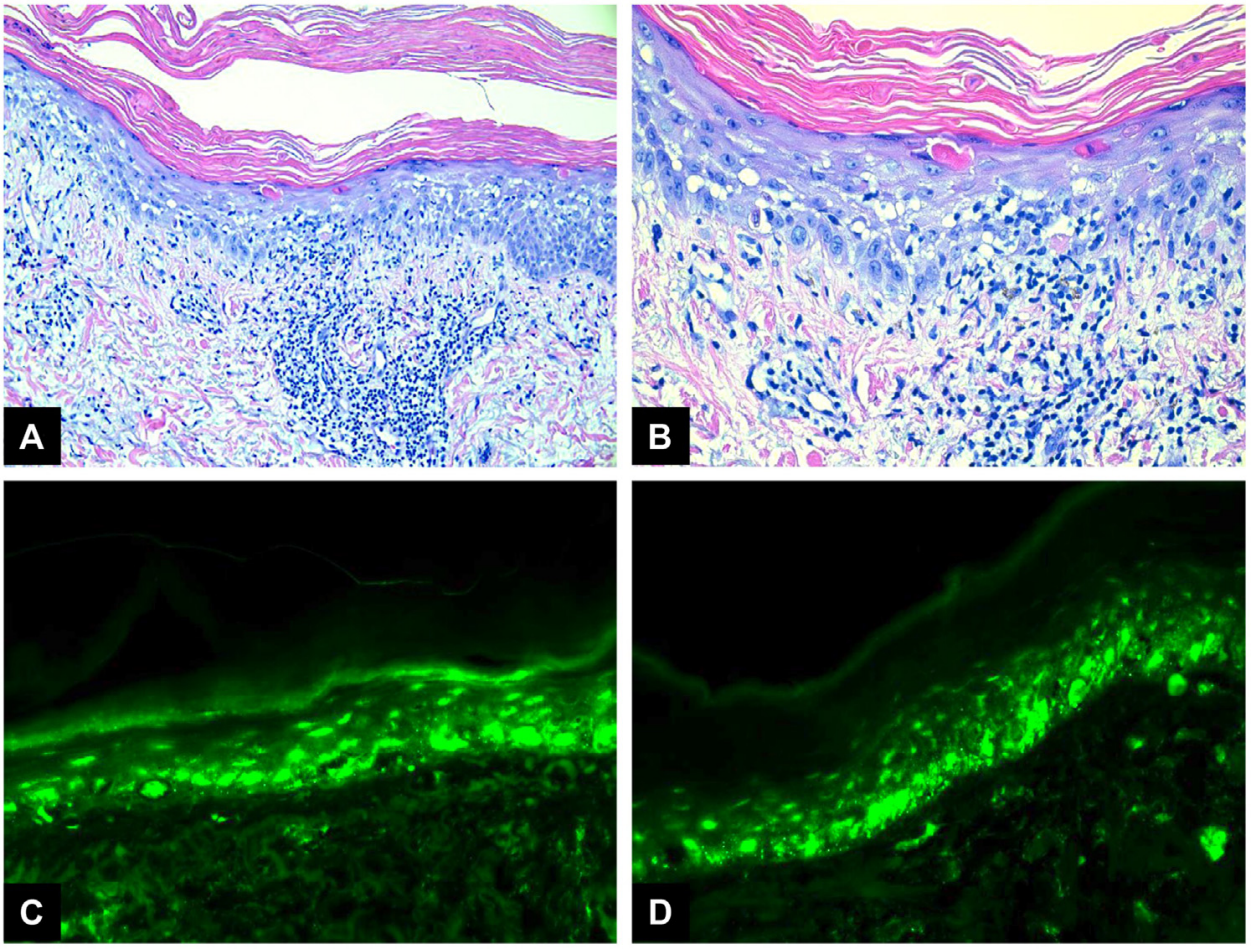
**A** and **B,** Punch specimen histopathology from patient showing interface vacuolar lymphocytic infiltrate with prominent scattered apoptotic keratinocytes. **C** and **D,** Immunofluorescence reveals a fine granular IgG deposition along epidermal basement membrane.

**Fig 3:**
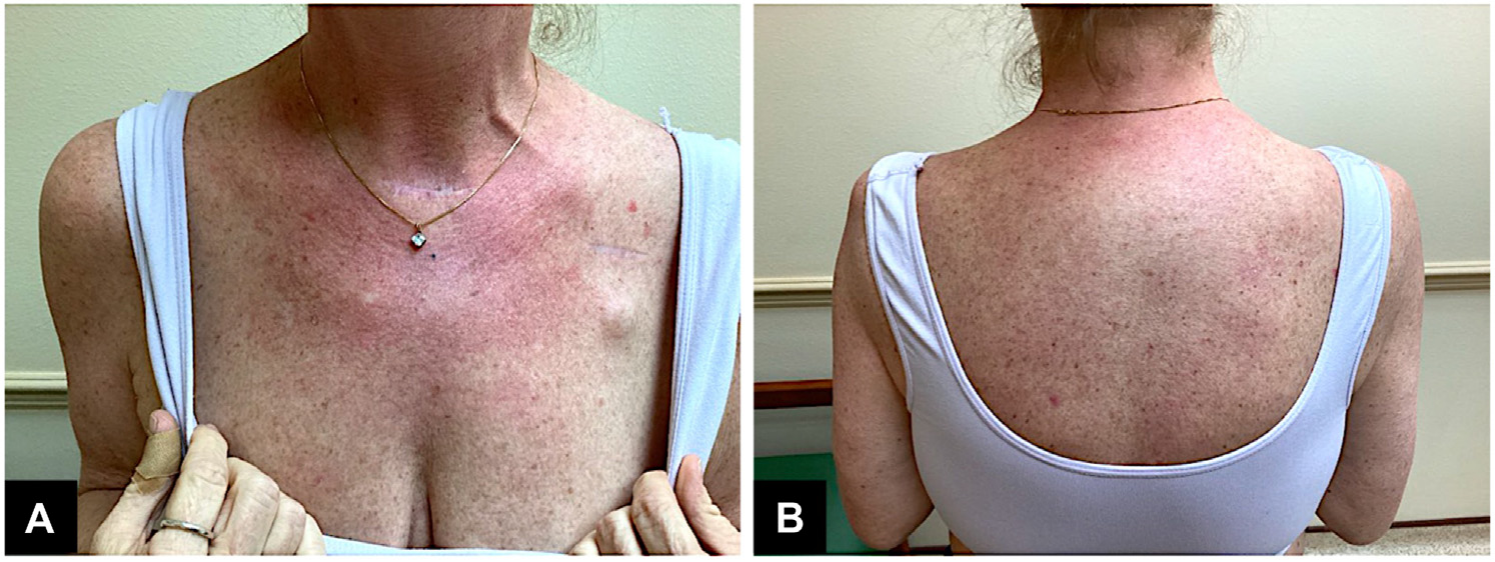
**A** and **B,** Patient presentation on the chest and upper back, respectively, at 8-week follow-up.

A potassium hydroxide preparation was negative for fungal hyphal elements. A punch biopsy was then obtained from the right anterolateral proximal upper arm and upper sternum and revealed vacuolar interface dermatitis with prominent scattered apoptotic keratinocytes and peri-adnexal and perivascular lymphohistiocytic inflammation (Fig 2). An autoimmune panel was notable for anti-Sjögren's-syndrome-related antigen A (anti-Ro/SSA) of 2.1 (normal range [0.0-0.9]), anti-Sjögren's syndrome B antibody (anti-La/SSB) of 0.6 (normal range [0.0-0.9]), antinuclear antibody of 7 (negative <5, equivocal 5-9, positive >9), and anti-histone antibodies of 0.7 (negative <1.0; normal range [0.0-0.9]). Immunofluorescence revealed a fine granular IgG deposition along the epidermal basement membrane zone and toward the skin strata's lower third, giving a characteristic dusting pattern. These data supported DI-SCLE. The patient was successfully treated with immediate withdrawal of the culprit drug (osimertinib) followed by initiation of a prednisone taper and topical triamcinolone ointment after biopsies were obtained. Hydroxychloroquine 200 mg twice daily was initiated for steroid-sparing effect. The patient discontinued the use of all medications prescribed by her 8-week follow-up. Her dermatitis had significantly improved (Fig 3).

## Discussion

DI-SCLE is a distinct subset of cutaneous lupus. Some cases of DI-SCLE have been associated with elevated anti-Ro/SSA.^5^ DI-SCLE's multifactorial pathogenesis involves genetic predisposition, drug transformation, and maladaptive immune responses. Some immunotherapy agents such as Cytotoxic T-lymphocyte-associated protein (CTLA-4), programmed cell death protein 1 (PD-1), and programmed death-ligand 1 (PD-L1) have been implicated in DI-SCLE cases.^6^, ^7^, ^8^

The patient had a cutaneous eruption 2 weeks after the initiation of osimertinib. Skin lesion biopsies revealed findings consistent with SCLE. Our case had an elevation of anti-Ro/SSA and a negative anti-histone antibody. Given the timing and onset of the eruption, along with the clinical, histopathologic, and serologic presentation, a diagnosis of DI-SCLE likely secondary to osimertinib, an EGFR TKI, is favored.

In conclusion, we describe a cutaneous AE likely induced by initiating osimertinib, a third-generation EGFR TKI. Given the clinical importance of this therapy in treating NSCLC, it is imperative to evaluate for potential rare AEs, such as DI-SCLE, which can impact a patient’s oncologic treatment course and outcomes. This case underscores the importance of prompt diagnosis and management in cases involving EGFR TKIs and other targeted therapies.

## Conflicts of interest

None disclosed.
